# Editorial Comment to A case of miliary tuberculosis following transurethral surgery and prostate biopsy after intravesical bacillus Calmette‐Guerin immunotherapy

**DOI:** 10.1002/iju5.12387

**Published:** 2021-11-11

**Authors:** Shingo Hatakeyama, Chikara Ohyama

**Affiliations:** ^1^ Department of Advanced Blood Purification Therapy Hirosaki University Graduate School of Medicine Hirosaki Japan; ^2^ Department of Urology Hirosaki University Graduate School of Medicine Hirosaki Japan

Intravesical bacillus Calmette–Guerin (BCG) therapy is standard therapy for T1 high‐grade high‐risk non‐muscle‐invasive bladder.^1^, ^2^ Although miliary tuberculosis after BCG therapy is rare (<1%),^3^ it could be life‐threatening. Yoshiaki *et al*. reported miliary tuberculosis defined by transurethral surgery and prostate biopsy specimens.^4^ This is an informative study reporting the risk of developing systemic tuberculosis after intravesical BCG. Some reports suggest that BCG may persist in the bladder for a long time and cause late‐onset BCG infection.^5^ A previous study reported that BCG PCR from bladder biopsy was positive in 23.8% (5 of 21 biopsies) at 9 months and 4.2% (1 of 25 biopsies) at 12 months after the final BCG instillation.^5^ Although the number of patients was limited, we should know that the risk of systemic infection might be not negligible after intravesical BCG therapy.

The key point that we need to learn from this case is how to avoid such an unusual incidence. According to the present case, urine BCG PCR might be an option for patients (i) who need to undergo an invasive procedure and (ii) who had persistent aseptic pyuria. Although urine BCG PCR test is not included in clinical practice, it might be helpful to recognize the risk of severe systemic tuberculosis after BCG therapy.

The association between BCG persistence and antitumor response is not known. BCG persistence in the bladder for a long time may cause late‐onset BCG infection but that may cause a long‐term durable response. Further study is necessary to address the long‐term persistence of BCG may be beneficial or harmful for the patient with high‐risk non‐muscle‐invasive bladder.

## Conflict of interest

The authors declare no conflict of interest.

## References

[1] Kikuchi E , Hayakawa N , Fukumoto K , Shigeta K , Matsumoto K . Bacillus Calmette‐Guérin‐unresponsive non‐muscle‐invasive bladder cancer: its definition and future therapeutic strategies. Int. J. Urol. 2020; 27: 108–16. 31793703 10.1111/iju.14153

[2] Miyamoto T , Miyake M , Toyoshima Y *et al*. Clinical outcomes after intravesical bacillus Calmette‐Guérin for the highest‐risk non‐muscle‐invasive bladder cancer newly defined in the Japanese Urological Association Guidelines 2019. Int. J. Urol. 2021; 28: 720–6. 33734503 10.1111/iju.14545

[3] Brausi M , Oddens J , Sylvester R *et al*. Side effects of Bacillus Calmette‐Guérin (BCG) in the treatment of intermediate‐ and high‐risk Ta, T1 papillary carcinoma of the bladder: results of the EORTC genito‐urinary cancers group randomised phase 3 study comparing one‐third dose with full dose and 1 year with 3 years of maintenance BCG. Eur. Urol. 2014; 65: 69–76. 23910233 10.1016/j.eururo.2013.07.021

[4] Kurowaka Y , Kawai T , Miyakawa J *et al*. A case of miliary tuberculosis following transurethral surgery and prostate biopsy after intravesical bacillus Calmette‐Guerin immunotherapy. IJU Case Rep. 2022; 5: 45–7. 10.1002/iju5.12385 PMC8720731 35005471

[5] Durek C , Richter E , Basteck A *et al*. The fate of bacillus Calmette‐Guerin after intravesical instillation. J. Urol. 2001; 165: 1765–8. 11342972

